# Supplementary material for: Complexome profiling of the Chlamydomonas psb28 mutant reveals TEF5 as an early PSII assembly factor
Source: Plant Cell. 2025 Mar 17;37(6):koaf055. doi: 10.1093/plcell/koaf055 (PMC12164590; doi:10.1093/plcell/koaf055)
Supplement: koaf055_Supplementary_Data [file koaf055_supplementary_data.zip › psb28_interactive.zip!/plots/intensity/qualityPlots/peptides/FAP380_Cre01.g010400.t1.2.html]

### Cre01.g010400.t1.2

Trivial name: FAP380  
SeqCoverage [median]: 10.20  
Mass: 20.40 kDa  
  
 

| Sequence | MissedCleavage | Mass [Da] | wtIntensity | muIntensity | Score |
| --- | --- | --- | --- | --- | --- |
| ALETYVK | 0 | 822.45 | 25397.97 | 44536.54 | 138.07 |
| FKSMDEFVAYLK | 1 | 1476.73 | 8419.96 | 27641.73 | 89.43 |
| SMDEFVAYLK | 0 | 1201.57 | 9058.20 | 18591.65 | 160.88 |
|  |  |  | 42876.1 | 90769.9 |  |
